# Barriers for access and utilization of dementia care services in Europe: a systematic review

**DOI:** 10.1186/s12877-025-05805-z

**Published:** 2025-03-10

**Authors:** Michele Sorrentino, Claudio Fiorilla, Michelangelo Mercogliano, Irene Stilo, Federica Esposito, Marcello Moccia, Luigi Lavorgna, Elena Salvatore, Maria Pia Sormani, Azeem Majeed, Maria Triassi, Raffaele Palladino

**Affiliations:** 1 Department of Public Health, University “Federico II” of Naples, Naples, Italy; 2 PhD National Programme in One Health Approaches to Infectious Diseases and Life Science Research, Department of Public Health, Experimental and Forensic Medicine, University of Pavia, 27100 Pavia, Italy; 3 Department of Advanced Biomedical Sciences, Federico II University of Naples, Via Pansini 5, 80131 Naples, Italy; 4 Multiple Sclerosis Unit, Policlinico Federico II University Hospital, Via Pansini 5, 80131 Naples, Italy; 5 Department of Advanced Medical and Surgical Sciences, University of Campania “Luigi Vanvitelli”, Via Pansini 5, 80131 Naples, Italy; 6 Department of Health Sciences, University of Genova, Genova, Italy; 7 Department of Primary Care and Public Health, School of Public Health, Imperial College, London, UK; 8 Interdepartmental Research Center in Healthcare Management and Innovation in Healthcare (CIRMIS), 80131 Naples, Italy

**Keywords:** Alzheimer, Dementia, Barriers, Systematic review

## Abstract

**Background:**

Dementia is a group of chronic diseases characterised by cognitive impairment that progressively disrupts daily functioning and requires increasing levels of healthcare, social support, and long-term care. Support for people with dementia can be provided by formal support systems although most of the care process relies upon informal care givers. Despite the availability of formal support systems and healthcare workers, the utilization of dementia care services remains suboptimal. Factors such as non-compliance, lack of awareness, and poor care coordination contribute to this issue. Understanding these barriers is crucial for improving service utilization and alleviating the economic burden on families and national health systems.

**Methods:**

This systematic review analysed the literature, published from 2013 to 2023, on barriers in Alzheimer and other dementia healthcare system, conducted on people living with a dementia, their caregivers, or healthcare workers in dementia care settings in Europe, following PRISMA guidelines. Searches in PubMed, Embase, PsycINFO, Health Technology Assessment Database, and Web of Science used terms related to Alzheimer’s, dementia, and access barriers. Rayyan AI supported full-text review, with quality assessed via the Mixed Methods Appraisal Tool.

**Results:**

Over 1298 articles, 29 studies met the inclusion criteria. These studies highlighted several barriers to dementia care, categorised into information, organizational, cultural, stigma-related, financial, and logistical challenges. Informational and educational barriers included a lack of awareness and knowledge among caregivers. Organizational barriers involved poor care coordination and unclear access procedures. Cultural and stigma-related barriers were linked to societal attitudes towards dementia. Financial barriers were associated with the high costs of care, and logistical barriers included limited availability and accessibility of support services.

**Conclusions:**

To enhance the quality of life for individuals living with dementia, it is crucial to address these identified barriers through tailored interventions and management programs. Improving care coordination, communication, and training for healthcare professionals, alongside reducing systemic delays, are essential steps toward more effective dementia care. Easing the burden of care with tailored interventions and management programmes is mandatory to improve the quality of life of persons living with dementia and their families.

## Introduction

Dementia includes a heterogeneous group of chronic diseases [1], characterised by cognitive dysfunction, impairments in activities of daily living and a high demand for health, social, and long-term care services [2]. In the European Union alone, nearly 14 million people already live with dementia [3]. Therefore, dementia has emerged as a “pandemic” in an ageing society [4], putting significant pressure on healthcare systems [5].

Care for people living with dementia is provided through formal support systems, including healthcare workers (HCWs) and care centres. However, a significant burden of care relies on both formal and informal caregivers [6], imposing a substantial economic burden on families due to direct out-of-pocket and informal care costs [7]. It is estimated that dementia care costs 392 billion EUR per year in Europe, with a cost per person of circa 27,815 [3]. Since the prevalence of dementia is growing steadily and is anticipated to double by 2050 [3], following the improvement in life expectancy and overall medical treatments [8], the costs for dementia care and the adherence to offered services are two pivotal factors for the sustainability of costs for national health systems.

Despite the availability of support services, their utilization is still partial, particularly for community services such as home support, day care, respite care, and counselling [9]. Several factors contribute to this low utilization, such as non-compliance with care and medication schedules, lack of awareness and knowledge among caregivers, unfamiliarity with support services, time constraints, limited availability of support services, poor care coordination and unclear access procedures [9–15], with communication from healthcare systems often being confusing and inaccessible for informal caregivers [16]. Furthermore, informal caregivers often perceive healthcare workers (HCWs) as lacking adequate training in dementia care provision, leaving them insufficiently prepared to provide comprehensive support [15]. The utilization of services may also be hindered by caregivers’ concerns about preserving their pride and autonomy, fearing that seeking help implies failure [17]. Services designed for carers often fail to address these barriers [18], with some carers refraining from seeking necessary care due to overwhelming emotions, such as fear, sense of duty and guilt [19, 20].

Overall, dementia management in primary care remains suboptimal [21]. Primary care physicians (PCPs) are often the first medical contact for individuals with cognitive problems and their caregivers [22], but face several challenges in providing optimal care to people living with dementia, such as insufficient training, limited consultation time, and a lack of comprehensive guidelines [11, 23–25]. Furthermore, HCWs faced significant challenges during the COVID-19 pandemic, which caused substantial disruptions in healthcare systems [26, 27]. These disruptions have further exacerbated symptoms in people living with dementia, including increased stress, behavioral problems, social isolation, and depressive symptoms, thereby compromising their quality of life [28, 29], and increasing the care burden and psychological distress for family caregivers [30–32].

Due to the increasing prevalence and costs of dementia care systems, it is essential to improve the access to existing healthcare services. Several barriers have been identified for accessing to services offered for national health systems to improve or ease the burden of dementia care. This review aims to systematically analyse and comprehensively assess these barriers within European settings. Furthermore, the aim is to provide information to policymakers and stakeholders to improve the efficacy of healthcare services offer to people living with dementia.

## Methods

This systematic review used the PICO framework to define the inclusion criteria, focusing on populations formally diagnosed with dementia or Alzheimer’s disease, along with both formal and informal caregivers and healthcare professionals. The interventions/outcome considered in this review involved barriers in accessing or utilising healthcare resources. No specific comparison was made. It was conducted following the Preferred Reporting Items for Systematic Reviews and Meta-Analyses (PRISMA) guidelines [33].

### Eligibility criteria

For this review we decided to include only original research (e.g., cohort studies, cross-sectional studies, case-control studies, or qualitative investigations), focusing on European settings, and available in English. We included studies conducted on people formally diagnosed with Alzheimer’s and other dementias, their caregiver or HCWs, regardless of age, gender, race, and socioeconomic status. All the studies had to address at least one barrier related to the access to care in dementia settings. A broader resume of the criteria is displayed in Table 1.

**Table 1:** Eligibility criteria

Inclusion Criteria	Exclusion Criteria
*Population* • European settings AND • People living with dementia or Alzheimer’s Disease with a formal diagnosis AND/OR • Formal and Informal caregivers of people living with dementia AND/OR • Healthcare professionals and other stakeholders involved in dementia care *Intervention* • Assessment or search of: Barriers to dementia care *Other criteria* • Written in English AND • Original Research AND • Studies published in or after 2013	*Population* • Studies focused on neurological diseases other than dementia or Alzheimer’s Disease *Intervention* • Other study types do not meet specified criteria (e.g.; randomized controlled trials, case control, editorial, letters, conference paper). *Other criteria* • Full text not available

### Search strategy

The primary dataset was PubMed/MEDLINE. Further searches were performed in: Embase, PsycINFO (EBSCOhost), Health Technology Assessment Database, and Web of Science (Clarivate). These searches covered a time frame of 10 years (2013–2023), to provide insights into recent developments in dementia care, capturing current challenges and barriers faced by patients and caregivers, and offering an up-to-date perspective on the evolving landscape. The research string was designed to identify studies addressing barriers in European Alzheimer and other dementia healthcare system. The keywords, aligned the PICO framework, include the following terms: Population (P) (“Alzheimer Disease” OR “Dementia”) AND Intervention/Outcome (I/O) (“Barrier*” OR “Access” OR “Healthcare” OR “Health Care Utilization”) AND Geographical Area (S) (“Europ*” [MeSH]) AND Timeframe (T) (“2013/01/01“[PDAT]: “2023/12/31“[PDAT]). Additional relevant papers were manually added from reference lists of collected studies and reviews.

**Table 2:** Research string explain for each domain

Study Population (*P*)	“Alzheimer Disease” OR “Dementia”
AND	
Intervention (I)/ Outcome (O)	(“Barrier*” OR “Access” OR “Healthcare” OR “Health Care Utilization”)
AND	
Comparison (C)	Not applicable
AND	
Geographical Area (S)	“Europ*” [MeSH]
AND	
Timeframe (T)	“2013/01/01“[PDAT]: “2023/12/31“[PDAT]

### Data extraction and quality assessment

Five reviewers (MS, CF, MM, FE, IS) screened the titles and abstracts of extracted articles, identifying those that met the inclusion criteria using Rayyan Artificial Intelligence [34] a tool tailored for systematic review support. Rayyan streamlines the screening process by allowing reviewers to independently tag articles, apply inclusion and exclusion criteria, and perform blind screenings, which helps to identify those adhering to the inclusion criteria. When abstracts did not provide sufficient information to assess eligibility, a full-text review was undertaken. The same five reviewers (MS, CF, MM, FE, IS) independently evaluated the full texts to determine if each study met the inclusion criteria. Conflicts were resolved by the senior reviewer (RP). If selected, pivotal information was extracted and reported in Excel sheet. The quality of the included papers was evaluated using the Mixed Methods Appraisal Tool (MMAT), revised version [35]. This assessment tool evaluates various aspects of study quality based on the specific design of each study, taking into account the unique features of each type. The scores for the MMAT range from 0 to 100% depending on the study design criteria. Studies with lower scores (e.g., below the 50% threshold), indicating potential methodological limitations, are not automatically excluded; instead, they are carefully examined to assess how their quality might influence the overall findings. This approach ensures that the review captures a full range of evidence, while also being transparent about how studies with lower quality scores are interpreted and integrated into the final conclusions. In mixed methods studies, the overall quality of the combination cannot surpass the quality of its weakest component. Therefore, the overall quality score is determined by the lowest score among the study components. The reviewers evaluated each paper independently to provide an objective assessment of the study quality.

## Results

### Study selection and quality assessment

The main research search identified 1748 studies. After adapting and running the research string on secondary database, duplicates were removed using Rayyan, and a database of 1202 unique studies was compiled. Additionally, 4 studies were identified through reference mining of three systematic reviews [36–38]. Upon review of their titles and abstracts, 1129 studies were deemed irrelevant and excluded. Subsequently, 73 publications underwent a full-text review, resulting in the selection of 29 studies meeting the inclusion criteria. Grey literature was not considered, as well as conference papers, dissertations, letters, and editorials.

The 44 studies were excluded for the following reasons: 27 studies lacked assessments of barriers, 12 studies were excluded due to study design or type, 4 studies were not set in a European nation, and 1 study was not focused on people living with dementia.

**Fig. 1:**
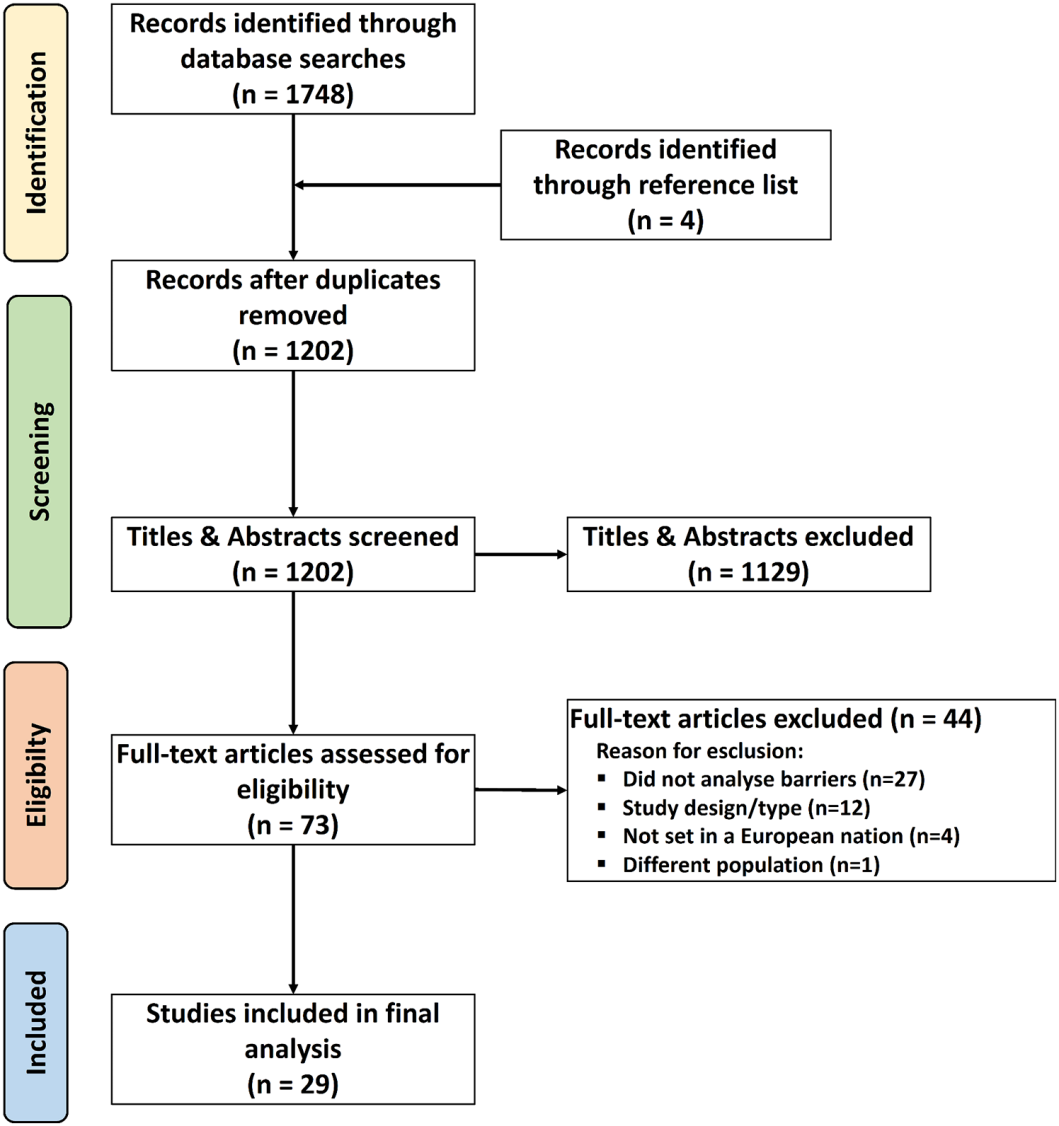
PRISMA Flow diagram of literature search, abstract screen, full article assessment for exclusion and inclusion criteria with most common reasons for exclusion

A visual representation of this selection process and the reason for exclusion is provided in the PRISMA diagram (Fig. 1).

The quality of each article was assessed using the MMAT tool to evaluate the rigor and reliability of findings related to barriers in dementia care. Most studies received a quality score of 100%, meeting all criteria for methodological rigor, including well-defined study design, transparent data collection, and thorough reporting. Articles with scores below 100%—particularly those with scores of 30% or 40%—were mixed methods or quantitative studies that showed limitations, such as potential sampling and non-response biases or insufficient methodological detail. These scores indicate variability in study quality, with the highest-scoring studies providing stronger evidence on identified barriers, while lower-scoring studies contributed insights with some caution regarding their reliability due to noted limitations.

### Study characteristics

The characteristics of the articles included are summarised in Table 3. 29 studies, spanning from 2013 to 2022 [39–67] were selected for this review. The publication years were: two studies in 2013 [39, 40], one study in 2014 [41], one in 2015 [42], one in 2017 [43], three in 2018 [44–46], three in 2019 [47–49], eight in 2020 [50–57], eight in 2021 [58–65], and two in 2022 [66, 67]. Some studies were conducted during the COVID-19 pandemic with varying degrees of focus on COVID-19: explicitly focused on it (*n* = 4) [55, 59, 60, 63, 64], did not focus on it (*n* = 3) [61, 66, 67], did not mention it (*n* = 2) [58, 65].

Most of the studies were conducted primarily in the United Kingdom (UK) (*n* = 13) [39–41, 44, 47, 49, 52, 54, 55, 59, 60, 64, 65], across various European countries, including multi-country studies under the Actifcare project (*n* = 2) [46, 48] and the Intermediate care for dementia in Europe (*n* = 1) [67]. Several studies included two countries: Italy and the Netherlands (*n* = 1) [43], Switzerland and Liechtenstein (*n* = 1) [61] and the UK and the Netherlands (*n* = 1) [58], while others were conducted in single countries such as in Germany (*n* = 2) [42, 56], in Norway (*n* = 2) [45, 50], Spain (*n* = 1) [51], Denmark (*n* = 1) [53], Finland (*n* = 1) [62], Poland (*n* = 1) [63], Portugal (*n* = 1) [66], and Ireland (*n* = 1) [57].

The methods employed varied across the studies. Qualitative studies (*n* = 24) utilised various techniques, including interviews (*n* = 13) [39–41, 44, 47, 48, 50, 55–57, 59, 61, 64], focus groups (*n* = 3) [42, 43, 46], both interviews and focus groups (*n* = 7) [45, 49, 52, 54, 62, 65, 66], and a combination of survey and focus groups (*n* = 1) [51]. Mixed-methods studies (*n* = 2) included surveys (*n* = 1) [67] and a combination of surveys and interviews (*n* = 1) [58]. Additionally, three quantitative studies (*n* = 3) employed a survey [53, 60, 63].

The reviewed studies encompassed various populations, with sample size ranging from 8 to 583. The focus of most studies was on HCWs (*n* = 9) [43–45, 47, 51, 53, 61, 62, 67], while others concentrated solely on people living with dementia (*n* = 1) [52], and on caregivers (*n* = 5) [41, 54, 56, 63, 64]. Both caregivers and HCWs participated in six studies [42, 49, 50, 57–59]. Some studies (*n* = 5) included both people living with dementia and caregivers [39, 40, 48, 55, 60]. Furthermore, several studies (*n* = 3) included people living with dementia, caregivers, and HCWs [46, 65, 66].

The studies assessed different care settings, focusing on either a single setting or multiple settings. The majority of studies (*n* = 11) focused on home-based care [39, 41, 44, 50, 53–56, 60, 63, 64], primary care (*n* = 2) [62, 66], clinical or hospital-based care (*n* = 3) [47, 51, 65], residential or long-term care facilities (*n* = 3) [43, 45, 61], and community-based care (*n* = 2) [52, 57]. Furthermore, multiple settings were explored in several studies: for instance, there were studies that combined home-based care with community-based care (*n* = 1) [48], while others combined home-based care with respite care (*n* = 3) [46, 58, 59]. Additionally, some studies (*n* = 3) examined a combination of home-based care, residential or long-term care facilities, and community-based care [40, 42, 67], whereas one study investigated a mix of home-based care, respite care, and clinical or hospital-based care (*n* = 1) [49].

**Table 3:** Studies characteristics

First Author, Year, (cit.)	Nation of study	Study Design	Methods	Population	Sample	Setting	Identified Barriers	Quality score
Dickinson C. et al., 2013 [39]	UK	Qualitative study	Interview	People living with dementia and Caregivers	46 (29 Caregiver; 17 people living with dementia)	- Home-Based Care	- Informational and educational	100%
Górska S. et al., 2013 [40]	UK	Qualitative study	Interview	People living with dementia and Caregivers	31 (19 Caregiver; 12 people living with dementia)	- Home-Based Care - Community-Based Care - Residential or Long-Term Care Facilities	- Organisational - Informational and educational	100%
Jutlla K. [41], 2014	UK	Qualitative study	Interview	Caregivers	12	- Home-Based Care	- Cultural and stigma-related	100%
Stephan A. et al., 2015 [42]	Germany	Qualitative study	Focus Group	HCWs and Caregivers	30 (13 HCWs; 17 Caregivers)	- Home-Based Care - Community-Based Care - Residential or Long-Term Care Facilities	- Organisational - Cultural and stigma-related	100%
Mariani E. et al., 2017 [43]	Italy, the Netherlands	Qualitative study	Focus Group	HCWs	19	- Residential or Long-Term Care Facilities	- Organisational - Financial	100%
Kupeli N. et al., 2018 [44]	UK	Qualitative study	Interview	HCWs	14	- Home-Based Care	- Organisational - Informational and educational - Logistical	100%
Midtbust M. et al., 2018 [45]	Norway	Qualitative study	Focus Group / Interview	HCWs	20	- Residential or Long-Term Care Facilities	- Organisational	100%
Stephan A. et al., 2018 [46]	Germany, Ireland, Italy, the Netherlands, Norway, Portugal, Sweden and the UK	Qualitative study	Focus Group	People living with dementia and HCWs and Caregivers	261 (114 HCWs; 96 Caregiver; 51 people living with dementia)	- Home-Based Care - Respite Care	- Organisational	100%
Burgon C. et al., 2019. [47]	UK	Qualitative study	Interview	HCWs	19	- Clinical or Hospital-Based Care	- Organisational - Informational and educational	100%
Kerpershoek L. et al., 2019 [48]	Netherlands, Germany, the UK, Ireland, Sweden, Norway, Portugal, Italy	Qualitative study	Interview	People living with dementia and Caregivers	85	- Home-Based Care - Community-Based Care	- Organisational, - Informational and educational - Cultural and stigma-related - Financial - Logistical	100%
Oliveira D. et al., 2019 [49]	UK	Qualitative study	Focus Group / Interview	HCWs and Caregivers	73 (46 HCWs; 27 Caregiver)	- Home-Based Care - Respite care - Clinical or Hospital-Based Care	- Organisational - Informational and educational - Financial - Logistical	100%
Czapka E. et al., 2020 [50]	Norway	Qualitative study	Interview	HCWs and caregivers	14 (6 HCWs, 8 caregivers)	- Home-Based Care	- Cultural and stigma-related	100%
Minaya-Freire A. et al., 2020 [51]	Spain	Qualitative study	Survey / Focus group	HCWs	10	- Clinical or Hospital-Based Care	- Organisational, - Informational and educational - Logistical	100%
Mitchell G. et al., 2020 [52]	UK	Qualitative study	Focus Group / Interview	People living with dementia	20	- Community-Based Care	- Cultural and stigma-related	100%
Nielsen T. et al., 2020 [53]	Denmark	Quantitave study	Survey	HCWs	41	- Home-Based Care	- Cultural and stigma-related	100%
Hossain MZ et al., 2020 [54]	UK	Qualitative study	Focus Group / Interview	Caregivers	27	- Home-Based Care	- Cultural and stigma-related, educational	100%
Giebel C et al., 2020 [55]	UK	Qualitative study	Interview	People living with dementia and Caregivers	342 (285 Caregiver; 61 PWLD)	- Home-Based Care	- Logistical	100%
Monsees J et al., 2020 [56]	Germany	Qualitative study	Interview	Caregivers	8	- Home-Based Care	- Educational	100%
Ryan L et al., 2021 [57]	Ireland	Qualitative study	Interview	HCWs and Caregivers	34 (14 HCWs; 20 Caregiver)	- Community-Based Care	- Cultural and stigma-related	100%
Giebel C. et al., 2021 [58]	UK, the Netherlands	Mixed methods study	Survey / Interview	HCWs and Caregivers	116 (13 HCWs; 103 Caregiver)	- Home-Based Care - Respite Care	- Informational and educational - Cultural and stigma-related	30%
Giebel C. et al., 2021 [59]	UK	Qualitative study	Interview	HCWs and Caregivers	21 (14 Caregiver; 7 people living with dementia)	- Home-Based Care - Respite Care	- Organisational	100%
Giebel C. et al., 2021 [60]	UK	Quantitave study	Survey	People living with dementia and Caregivers	13	- Home-Based Care	- Organisational	100%
Hirt J. et al., 2021 [61]	Switzerland, Liechtenstein	Qualitative study	Interview	HCWs	12	- Residential or Long-Term Care Facilities	- Organisational	100%
Kulmala J. et al., 2021 [62]	Finland	Qualitative study	Focus Group / Interview	HCWs	27	- Primary Care - Public Health Settings	- Organisational - Informational and educational	100%
Rusowicz J. et al., 2021 [63]	Poland	Quantitative study	Survey	Caregivers	85	- Home-Based Care	- Organisational	30%
Sriram V. et al., 2021 [64]	UK	Qualitative study	Interview	Caregivers	23	- Home-Based Care	- Logistical	100%
Wheatley A. et al., 2021 [65]	UK	Qualitative study	Focus Group / Interview	HCWs, people living with dementia and Caregivers	177 (129 HCWs; 31 Caregiver; 17 people living with dementia)	- Clinical or Hospital-Based Care	- Organisational	100%
Balsinha C. et al., 2022 [66]	Portugal	Qualitative study	Focus Group / Interview	HCWs, people living with dementia and Caregivers	40 (22 HCWs; 10 Caregiver; 8 people living with dementia)	- Primary Care	- Organisational - Informational and educational - Cultural and stigma-related	100%
Dibao-Dina C. et al., 2022 [67]	Bosnia, Croatia, Georgia, Greece, France, Hungary, Ireland, Israel, Italy, Latvia, Poland, Portugal, Romania, Switzerland, the UK, Ukraine	Mixed methods study	Survey	HCWs	583	- Home-Based Care - Community-Based Care - Residential or Long-Term Care Facilities	- Financial	40%

### Identified barriers

Multiple barriers to accessing and utilising dementia care services have been identified. These barriers were broadly categorised into organisational barriers (*n* = 17) [40–44, 46–51, 53, 59–63, 65, 66] which involve issues of coordination, communication, and stability within organisations that hinder effective service delivery; information and educational barriers (*n* = 11) [39, 40, 44, 46, 48, 49, 51, 54, 56, 62, 66] related to a lack of adequate information, knowledge, and skills in managing dementia care; cultural and stigma-related barriers (*n* = 10) [42, 48, 52, 54, 57, 58, 66] involving social prejudices, misconceptions, and a lack of cultural understanding that reduce the accessibility and efficacy of care; logistical barriers (*n* = 6) [44, 48, 49, 51, 55, 64] which include practical challenges in accessing services, such as waiting times, continuity, and difficulties in service use and financial barriers (*n* = 4) [43, 48, 49, 67], encompassing economic constraints and complex administrative processes that limit access to necessary services.

#### Organisational barriers

Organizational and coordination challenges in dementia care are widespread across Europe, with high staff turnover, inconsistent care strategies, poor team communication, and limited community service information notably affecting systems in several European countries. These issues impact Northern (the UK, Norway), Western (Germany, Liechtenstein, Switzerland, and the Netherlands), and Southern Europe (Portugal, Italy, and Spain) [40, 42–45, 51, 61, 66]. Fragmented services, seen across much of Europe, including Northern (Ireland, the UK, Sweden, and Norway), Western (Germany and the Netherlands), and Southern Europe (Portugal and Italy), disrupt continuity of care, burdening both people with dementia and their caregivers [46–48]. Inadequate resources and inequitable services further challenge healthcare workers in the Northern countries (the UK and Finland), while unclear role definitions and poor team coordination add to the strain in Southern (Portugal) and Northern countries (the UK) [47, 60, 62, 65]. The COVID-19 pandemic intensified these issues, limiting access to health and social services, especially in the Northern (the UK) and Eastern countries (Poland) [60, 63].

#### Information and educational barriers

Access to information on dementia care is limited in several European countries, which include parts of Northern (Ireland, the UK, Norway, and Sweden), Western (Germany and the Netherlands), and Southern Europe (Italy and Portugal), HCWs report insufficient resources and support [46, 54, 56]. Health literacy challenges also persist in Northern (the UK) and Western countries (the Netherlands) [49, 58], and limited dementia care skills are reported in Southern Europe (Portugal) [66]. Advance care planning remains difficult to coordinate in Northern Europe (the UK) [39], as does post-diagnostic support [40]. Low levels of health literacy, limited skill-sharing, and inadequate training for healthcare workers add further complications in several Northern European countries (Ireland, the UK, Norway, Sweden), Western European countries (Germany and Netherlands), and Southern European countries (Portugal and Italy) [44, 48, 51], as well as in Northern (Finland) and Southern countries (Portugal) [62, 66].

#### Cultural and stigma-related barriers

Social stigma, misconceptions, and public perception issues in dementia care make it challenging for individuals with dementia and their caregivers to accept and evaluate the appropriateness of available support services, particularly in community-based and home-based care settings in Northern (Ireland and the UK) and Western Europe (Germany and the Netherlands) [42, 52, 57, 58]. Moreover, recognizing and respecting the autonomy and social health needs of individuals with dementia is often limited wthinin primary care systems in Southern Europe (Portugal), adding complexity to the provision of comprehensive and person-centered care [66]. These barriers also create challenges for healthcare workers across several European countries, such as in Northern (the UK, Ireland, Norway, and Sweden), Western (Germany and Netherlands), and Southern Europe (Portugal and Italy), where fragmented services and limited coordination add to the strain in home-based and community-based care settings [48]. In Northern Europe (the UK, Denmark, Norway), where diverse immigrant communities are part of the social fabric, dementia care faces unique cultural and communication challenges. In the UK, Sikh caregivers struggle with community and cultural norms that hinder access to health and social care services [41]. Additionally, HCWs encounter difficulties in understanding the cultural and religious values of Bangladeshi communities [54]. In Denmark, HCWs working with minorities from the Middle East (particularly Turkey and Iran), Eastern Europe (mainly the former Yugoslavia and Poland), and Pakistan minorities report communication difficulties, strong cultural norms, and stigma within these groups [53] A similar situation is present in Norway for minority groups such as Somalis, Pakistanis, Turks, Poles, Croatians, and Indians [50].

#### Financial barriers

Economic constraints significantly impact families’ ability to afford dementia care services across several European countries, such as in Northern (Ireland, the UK, Norway, and Sweden), Western (Germany and the Netherlands), and Southern Europe (Portugal and Italy) [48, 49]. Financial strain is worsened by complex administrative processes in several countries, adding to the burden on families. These include Southern Europe (Italy, Greece, Portugal), Western Europe (the Netherlands, France, Switzerland), Eastern Europe (Bosnia, Croatia, Georgia, Hungary, Latvia, Poland, Romania, Ukraine), Northern Europe (Ireland and the UK) [43, 67].

#### Logistical barriers

Logistical barriers also affect both caregivers and HCWs. Caregivers in Northern Europe (the UK) report personal challenges that complicate their responsibilities [49]. During the pandemic, these challenges grew as accessing formal care and respite services became even more difficult, underscoring the need for additional support in using telecare solutions [55, 64]. HCWs in several Northern European countries (the UK, Ireland, Norway, and Sweden), Western (Germany and Netherlands), and Southern European countries (Portugal and Italy) face difficulties in maintaining consistent contact with patients due to time constraints, work overload, and long wait times [44, 48, 51].

## Discussion

This systematic review analyzed 29 studies published between 2013 and 2022 to identify key barriers to accessing and utilizing dementia care services across Europe. The findings reveal a range of significant barriers spanning organizational, informational, educational, cultural, stigma-related, financial, and logistical domains, with additional unique challenges faced by minority groups.

Dementia care in Europe faces substantial challenges rooted in the diversity of healthcare systems, cultural models, and economic resources. The studies’ broad geographic representation—most prominently from the UK, along with contributions from multiple European countries—highlights the widespread relevance of these issues. This range illustrates the pressing need for international collaboration and coordinated efforts to address the disparities in dementia care access and impact across different populations [68].

The complexity of the European dementia care landscape calls for strategies that consider the distinct socioeconomic and cultural contexts of each region. Tailored, region-specific approaches are essential to ensure equitable access to dementia care and support across diverse communities.

### Organisational barriers

Organizational barriers are common across Europe and create significant challenges in providing effective care for people living with dementia and their caregivers. These barriers are characterised by fragmented services, inadequate healthcare resources, and poor coordination within care teams continue to strain systems in numerous countries, including Northern, Western and Southern Europe [42–46, 48, 49, 51, 61, 66], significantly increasing healthcare costs [68–70] and the risk of comorbidity [71–73]. In Northern Europe, particularly in countries like the UK and Finland, insufficient healthcare resources and inequitable services place additional strain on HCWs [47, 60, 62, 65]. Undefined roles, poor coordination, and continuity within care teams were identified as significant obstacles for families and HCWs in Portugal and the UK [40, 66], as well as limited capacity and capability in primary care in the UK [65], compounded by high staff turnover and unclear roles, lead to instability within care teams, hindering continuity and the development of trusting relationships between caregivers and patients [74]. The COVID-19 pandemic has exacerbated these problems, further limiting access to support services, and deepening existing inequalities, especially in Poland and in the UK [60, 63]. It has also highlighted the need for psychological and social support to address these challenges [75] and emphasised the necessity for integrated, patient-centered care pathways, to overcome care fragmentation [76, 77]. Strong coordination between multiple service providers can improve population health outcomes [74, 78] and deliver health services in a cost-effective manner [79, 80].

### Information and educational barriers

Information and educational barriers are also critical issues in several countries, such as Germany, Ireland, Italy, the Netherlands, Norway, Portugal, Sweden and the UK, as insufficient dissemination of knowledge among families and HCWs, along with poor health literacy, hinder advance care planning and access to coordinated post-diagnostic support [46, 49, 58]. The lack of clear and accessible information about available services exacerbates the situation, making it difficult for families to find the support they need [39, 40, 44, 48, 49, 51, 54, 56, 62, 66]. Education and support services for people living with dementia and their caregivers have positive effects, boosting their confidence, reducing stress and depression, and improving overall well-being [81–84]. However, educational programs are complex interventions influenced by multiple factors affecting their delivery, outcomes, and adaptation in practice [85]. HCWs encounter numerous barriers, including low health literacy among patients [58], limited knowledge-sharing opportunities [44, 48, 51], and inadequate training on disease management, especially noted in Finland and other countries [62, 66].

To ensure effective training, it is crucial for HCWs to have dedicated time for education and skill development [86]. However, organizations often struggle with resources such as time, finances, and staff availability, which are necessary to effectively implement learning initiatives [87–88].

### Cultural and stigma-related barriers

Cultural and stigma-related barriers significantly impact the acceptance of help and the appropriateness of services for people living with dementia and their caregivers, affecting their quality of life. In many cultures, dementia is still viewed as a shameful condition or a normal part of aging rather than a manageable disease [89]. Social prejudices and misconceptions regarding dementia limit the autonomy of people living with dementia and hinder HCWs’ efforts to provide effective care, particularly in Northern, Western and Southern countries [42, 52, 58, 66]. Stigmatization, often resulting from a lack of understanding and cultural taboos around dementia, leads families and people living with dementia to avoid seeking help, exacerbating isolation and difficulties in managing the disease, across Northern, Western and Southern European countries [48, 57]. This social isolation increases rates of loneliness and depression, further worsening mental health and well-being, and creating a cycle of exclusion and deteriorating conditions [90]. Cultural barriers also shape families’ expectations and experiences of dementia care, with taboos and misconceptions contributing to the stigma [91]. Fear of discrimination, social isolation, and judgment by the community can delay diagnosis, treatment uptake, and access to support services [92]. Addressing these issues requires increased dementia awareness to combat stigma and the perception of dementia as part of normal aging [93]. Research highlights the importance of recognising the diversity in sociocultural factors across cultures and societies and how these shape people’s understanding of dementia, as well as prevention and care management interventions [94]. Tailored interventions that center the experiences and voices of local communities, considering geopolitical and social contexts, are crucial for developing awareness and creating targeted responses, especially in low- and middle-income countries [95, 96]. HCWs must be aware of the stigma and fear attached to diseases such as dementia and be trained to address these sensitive issues in a way that alleviates fear and stigma for diagnosed individuals and their caregivers [97].

A crucial aspect that emerged is the specific difficulties faced by minority groups, who encounter additional barriers due to the lack of cultural sensitivity in healthcare services, stigma within communities, and communication difficulties [41, 50, 53, 54]. The lack of cultural understanding and adaptation by HCWs can lead to inadequate care and limited access for these groups [98], resulting in delays or general non-contact with PCPs about their symptoms [99]. These issues contribute to poor awareness of and cultural sensitivity towards different aspects of dementia care services [100–102]. A lack of language skills hinders contact with healthcare institutions [101]. Using interpreters during assessments only partially eliminates the language barrier, as interpreters often lack experience in dementia assessment and culturally and linguistically adapted assessment tools are scarce [103, 104]. Consequently, individuals from these groups often avoid accessing dementia care, highlighting the need for culturally sensitive approaches [105] and improved access to dementia services for all ethnic groups [100, 106, 107]. Addressing these barriers requires a multifaceted approach, including increasing cultural competency among healthcare workers, developing language-accessible resources, and engaging minority communities in conversations about dementia care. Culturally inclusive training for healthcare providers, such as learning to recognise and respect differing perspectives on dementia and aging, can facilitate trust and improve the experience of both people with dementia and their carers. The creation of multilingual materials and the employment of interpreters with specific training in dementia care could also enhance access for non-native speakers.

### Financial barriers

Financial barriers place a substantial burden on families across Europe, especially in Western, Northern and Southern Europe. The high costs of dementia care services, combined with complex administrative requirements, add significant stress to families already challenged by disease management [43, 48, 49, 67]. This financial strain adversely affects the mental and physical well-being of both patients and their caregivers [110].

These economic pressures can restrict access to essential health and social services [104, 111, 112] and high-quality treatments [113], resulting in suboptimal disease management and deteriorating health conditions. Insufficient public funding for care services and limited insurance coverage are often at the root of these financial challenges [6]. Policies and interventions that address financial disparities directly could improve health outcomes for older adults, offering much-needed relief to families and enhancing their ability to access vital care services [114–116].

### Logistical barriers

Logistical barriers, such as long waiting lists, transport difficulties, and a lack of available time for caregivers, make it challenging to provide continuous, quality care [44, 48, 49, 51, 55], across in especially in Western, Southern and Northern Europe countries in Europe. These barriers are often amplified in rural areas or regions with poor healthcare infrastructure [108–110], making access to dementia care even more difficult [110, 111]. Efficient healthcare, particularly in rural areas, relies heavily on cooperation and existing formal and informal networks between providers of health, social, and administrative services [112]. However, service systems are often overly complex and difficult to navigate from the caregiver perspective [20, 113]. Difficulties in accessing services can lead to delays in care [114], and caregivers may feel further stressed and overloaded when they are unable to obtain essential resources despite significant efforts [115].

The diversity of care settings studied in the reviewed research, including home-based care [39, 41, 44, 50, 53, 60, 63, 64], primary care [62, 66], clinical or hospital-based care [47, 51, 65], residential or long-term care facilities [43, 45, 61], and community-based care [52], underscores the potential for fragmentation in the care pathway, as different environments can lead to inconsistencies in the quality and continuity of care provided [116]. Integrated care systems allow for rapid response to the assessment and management of the care needs of people living with dementia [117], underscoring the urgent need for functional and integrated dementia care pathways to improve access to specialised care and minimize disruptions in care plans [117, 118], thereby promoting the well-being of individuals and ensuring seamless care across professional boundaries [119]. Such pathways should include access to specialised dementia care spaces and personalised care that is well-coordinated across different healthcare and social care settings [118].

## Policies

Recognising and addressing the various barriers identified in dementia care is crucial. By focusing on building person-centered healthcare systems, these barriers can be mitigated, enhancing the quality and continuity of care. Integrating culturally sensitive approaches, improving healthcare infrastructure, and ensuring seamless coordination among care providers will not only reduce the burden of dementia on patients and caregivers but also alleviate the strain on healthcare systems.

In this context, the European Union has made significant strides in supporting dementia care through various initiatives. These include political declarations and conferences that have elevated the profile of dementia as a public health priority at the European level, as well as funded programs and instruments to promote innovative solutions [120]. Building on this foundation, many European countries have implemented national dementia strategies aimed at early diagnosis, public awareness, comprehensive support services, and innovative research [121]. A milestone in this effort was the Glasgow Declaration of 2014, which called for the creation of a European Dementia Strategy and urged every country to establish national strategies [122].The momentum generated by these efforts has been reinforced through initiatives like Alzheimer Europe’s Strategic Plan 2021–2025 [123] and the Helsinki Manifesto [124] advocate for stronger political commitment and more comprehensive frameworks for dementia care, providing a roadmap for advancing care and addressing the challenges associated with dementia at both national and international levels.

Crucially, these policy advancements are complemented by initiatives that actively involve people living with dementia. For example, the European Working Group of People with Dementia (EWGPWD) [125] highlights the importance of inclusivity and ensures that the voices of those directly affected are integral to the decision-making process. By emphasising patient-centeredness and inclusivity in healthcare reforms is essential for creating more efficient, equitable, and responsive dementia care services in the future. Overall, this review highlights the need for more inclusive and differentiated research to fully understand and address the barriers to dementia care in diverse populations and settings.

## Strengths and limitations

To our knowledge, this is the first review summarising the main barriers to accessing and utilising dementia care services in Europe. This systematic review has several strengths that enhance the robustness of its findings. Firstly, it follows the Preferred Reporting Items for Systematic Reviews and Meta-Analyses (PRISMA) guidelines, ensuring a transparent and rigorous review process that minimizes bias and enhances reproducibility. Additionally, the review employs a comprehensive search strategy across multiple databases over a ten-year period, maximising the retrieval of relevant literature and providing a thorough overview of the topic. The involvement of multiple reviewers further enhances the reliability of study selection and reduces the likelihood of errors. Moreover, the systematic assessment of the methodological quality of included studies strengthens the validity of the review’s findings by identifying potential biases and limitations within individual studies.

The use of Rayyan AI for screening studies added significant value to the review process. Rayyan AI facilitated efficient and accurate title and abstract screening, allowing reviewers to quickly filter relevant studies while minimizing errors and inconsistencies. This AI tool’s functionality enabled better management of the large volume of studies retrieved, ensuring that relevant literature was included in the analysis without compromising quality.

Conducted across various European countries, the studies encompassed diverse roles, including people living with dementia, caregivers, and HCWs, analysing these groups both collectively and separately to yield robust findings.

Despite these strengths, there are several limitations to acknowledge. One limitation of this review is the exclusive focus on studies conducted within European settings, which may restrict the generalizability of findings to non-European contexts where healthcare structures, cultural attitudes, and resource allocations may differ significantly. While the study provides valuable insights into barriers specific to dementia care within European healthcare systems, it limits the scope of understanding to European-specific cultural, socioeconomic, and structural contexts. Hence, the challenges faced in non-European settings, where healthcare infrastructures, cultural attitudes toward dementia, and financial resources may differ significantly, are not captured. Multiple studies focused on more than one setting and often did not differentiate the results according to these settings, making it difficult to identify barriers specific to particular environments. Additionally, most studies did not specify the race and ethnicity of the participants, limiting the understanding of barriers across different racial and ethnic groups. Nonetheless, some studies did investigate the specific barriers faced by ethnic minorities, providing valuable insights.

A fundamental limitation of this review is the deliberate exclusion of randomized controlled trials (RCTs). RCTs, with their emphasis on testing interventions under controlled conditions, may not fully capture the complex, context-specific barriers that are the focus of this study. However, this choice was made to prioritize an in-depth assessment of real-world challenges, which aligns closely with research objectives. Future work in this area could benefit from combining qualitative and quantitative approaches to offer a more balanced perspective on both the barriers themselves and the effectiveness of interventions designed to address them. Furthermore, while this review has focused on identifying and analyzing key barriers to dementia care, it did not assess the effectiveness of specific strategies already in place to address these barriers. Future research should consider evaluating the impact of these existing strategies to provide a more comprehensive understanding of which interventions have been most successful in overcoming these challenges. This limitation reflects the prioritization set by our guidelines, which emphasized barrier identification over strategy evaluation.

Another notable limitation is the exclusion of barriers related to assistive technologies. While we acknowledge their growing significance in dementia care, the complexity and scope of this topic necessitate a dedicated review to comprehensively address the specific nuances and challenges involved. Furthermore, the reliance on assistive technologies surged during and after the COVID-19 pandemic, underscoring the urgent need for focused research to better understand their impact on access and utilisation of dementia care services in the future.

Finally, the restriction to studies published in English may introduce language and geographical biases, potentially overlooking valuable insights from non-English literature. The reliance on published literature may also lead to publication bias, as studies with positive results are more likely to be published, potentially skewing the overall findings.

## Conclusion

Dementia care in Europe presents common challenges across the region, which includes Northern, Southern, Eastern, and Western Europe. The intensity and specific nature of these challenges vary according to local contexts and characteristics. The continent’s cultural diversity and differences in national healthcare systems create unique complexities in managing dementia care, requiring flexible, context-specific solutions. Recognizing and addressing these barriers—including organizational, informational, educational, cultural, stigma-related, financial, and logistical obstacles—is essential for building a more inclusive and effective healthcare system. Improving service coordination, enhancing health literacy, raising awareness, and promoting cultural sensitivity are critical steps toward overcoming these challenges. It is crucial to consider the differences among European countries to develop adaptable policies that strengthen dementia care across the region while respecting each nation’s unique context. Policies that reduce care costs, streamline administrative processes, promote telecare technologies, and reduce waiting lists can ease the burden on families and improve care quality. Additionally, training healthcare workers in cultural competence and involving minority communities in designing services can ensure fair access and a higher standard of care for all. By focusing on person-centered healthcare systems that incorporate these considerations, it is possible to lessen the impact of dementia on patients and caregivers and reduce the pressure on healthcare systems. Emphasizing patient-centeredness and inclusivity in healthcare reforms is essential for creating more efficient, equitable, and responsive dementia care services across Europe in the future.

## Data Availability

No datasets were generated or analysed during the current study.

## Abbreviations

HCWs: Healthcare workers
COVID-19: CoronaVirus Disease 19
PCPs: Primary care physicians
UK: United Kingdom
EWGPWD: European Working Group of People with Dementia
